# Survivorship and clinical outcomes of proximal femoral replacement in non-neoplastic primary and revision total hip arthroplasty: a systematic review

**DOI:** 10.1186/s12891-021-04711-w

**Published:** 2021-11-08

**Authors:** Fabio Mancino, Vincenzo Di Matteo, Fabrizio Mocini, Giorgio Cacciola, Giuseppe Malerba, Carlo Perisano, Ivan De Martino

**Affiliations:** 1 Division of Orthopaedics and Traumatology, Department of Aging, Neurological, Orthopaedic and Head-Neck studies, Fondazione Policlinico Universitario Agostino Gemelli IRCCS, 00168 Rome, Italy; 2 Catholic University of the Sacred Heart, Largo Francesco Vito 1, 00168 Rome, Italy; 3 Adult Reconstruction and Joint Replacement Service, Division of Orthopaedics and Traumatology, Department of Aging, Neurological, Orthopaedic and Head-Neck studies, Fondazione Policlinico, Universitario Agostino Gemelli IRCCS, Largo Agostino Gemelli 8, 00168 Roma, RM Italy; 4 Orthopaedic Institute of Southern Italy “Franco Scalabrino”, Messina, Italy

**Keywords:** Proximal femoral replacement, Proximal femoral arthroplasty, Femoral revision, Femoral bone loss, Bone defect, Femoral reconstruction, Total hip arthroplasty, Revision hip arthroplasty

## Abstract

**Background:**

Several studies have evaluated the survivorship and clinical outcomes of proximal femoral replacement (PFR) in complex primary and revision total hip arthroplasty with severe proximal femoral bone loss; however, there remains no consensus on the overall performance of this implant. We therefore performed a systematic review of the literature in order to examine survivorship and complication rates of PFR usage.

**Methods:**

A systematic review of the literature according to the Preferred Reporting Items for Systematic Reviews and Meta-Analyses guidelines was performed. A comprehensive search of PubMed, MEDLINE, EMBASE, and the Cochrane Database of Systematic Reviews was conducted for English articles using various combinations of keywords.

**Results:**

In all, 18 articles met the inclusion criteria. A total of 578 PFR were implanted. The all-cause reoperation-free survivorship was 76.6%. The overall complication rate was 27.2%. Dislocation was the most common complication observed and the most frequent reason for reoperation with an incidence of 12.8 and 7.6%, respectively. Infection after PFR had an incidence of 7.6% and a reoperation rate of 6.4%. The reoperation rate for aseptic loosening of the implant was 5.9%. Overall, patients had improved outcomes as documented by postoperative hip scores.

**Conclusion:**

PFR usage have a relatively high complication rate, however, it remains an efficacious treatment option in elderly patients with osteoporotic bone affected by severe proximal femoral bone loss. Modular designs have shown reduced dislocations rate and higher survivorship free from dislocation. However, PFR should only be used as salvage procedure when no other reconstruction options are available.

## Background

Total hip arthroplasty (THA) is one of the most successful surgical procedures of the past 50 years. However, despite the overall success, revision THA remains a costly and challenging procedure to manage for the surgeon, especially in case of severe femoral and/or acetabular bone loss [1].

In the setting of femoral revision arthroplasty, significant bone loss continues to be problematic for healthcare professionals, potentially threatening the primary fixation and durability of the reconstruction. Reduced bone stock can be associated with septic and aseptic failure, periprosthetic fracture, osteoporotic fracture in the elderly with severe comminution or failed fracture fixation, and multiple revisions [2–7]. In case of severe proximal femoral bone loss multiple treatment options have been described in the literature, including structural allograft-prosthesis composite, impaction allografting, long revision stems, resection arthroplasty, and proximal femoral replacement (PFR) [3, 4, 8]. Proximal femoral replacement, also known as “megaprosthesis”, is a well-established limb salvage procedure for reconstruction of bone defects after the oncological resections of malignant bone neoplasms [9] and the encouraging outcomes have broadened the indications to the treatment of severe bone loss in non-oncologic conditions [10, 11].

PFR allows a faster recovery especially in elderly patients, and it avoids the disadvantages of bone grafting such as resorption, graft integration, and diseases transmissions [12, 13]. However, it is associated with an increased risk of infection and instability, secondary to the difficult healing of the abductor mechanism [14].

Multiple studies have described the outcomes of PFR in oncologic patients [9, 10], however, only few have described PFR usage in non-oncologic severe femoral bone loss associated with periprosthetic fracture, septic and aseptic revisions, or failed osteosynthesis. We therefore performed a systematic review of the literature in order to examine survivorship and complication rates of PFRs. Specifically, we aimed to examine (1) what is the survivorship from reoperation when PFRs are used? (2) what complications are most common in PFRs? (3) what is the cumulative incidence of hip dislocation with PFRs? (4) do PFRs provide adequate implant survivorship in line with alternative treatment methods in the management of severe femoral bone loss? and (5) what are their clinical outcomes?

## Methods

### Search strategy

This search was conducted in accordance with the Preferred Reporting Items for Systematic Reviews and Meta-Analyses guidelines [15]. The US National Library of Medicine (PubMed/MEDLINE), EMBASE, and the Cochrane Database of Systematic Reviews were queried for publications utilizing various combinations of the search terms “proximal femur replacement,” “proximal femur megaprosthesis,” “hip megaprosthesis,” “proximal femur arthroplasty,” “non-oncologic,” “bone loss,” “femoral reconstruction,” “bone defect,” “femoral revision,” in combination with the Boolean operators (AND, OR, *) since inception of database to January 2021. No limit was set with regard to the year of publication. Two authors (F.Ma. and V.D.M.) independently conducted all the searches and screened the titles and abstracts to identify relevant studies. Differences were resolved by consulting a third senior reviewer (I.D.M.). Only abstracts that evaluated the clinical outcomes and survivorship of non-oncologic patients with PFR following primary or revision THA were reviewed. If the title and abstract of each study contained insufficient information, the full manuscript was reviewed. An additional search was conducted by screening the references list of each selected article.

### Inclusion and exclusion criteria

Inclusion criteria were any original study in which a PFR was used in primary or revision THA in non-oncologic patients. Postoperative complications, clinical outcomes using validated patient reported scales and implant survivorship where reported. Exclusion criteria were case reports, surgical technique reports, review articles, expert opinions, letters to editors, biomechanical reports, instructional course lectures, studies on animals, cadaver or in vitro investigations, book chapters, abstracts from scientific meetings, unpublished reports, studies with less than 5 hips, studies with a mean follow-up less than 1 year, studies using the same database of patients, studies reporting the use of PFR in oncologic patients, and studies written in non-English language. If a duplicate population was noticed, the study with the longer mean follow-up was included to avoid including the same patients twice.

### Data extraction and collection

**Fig. 1:**
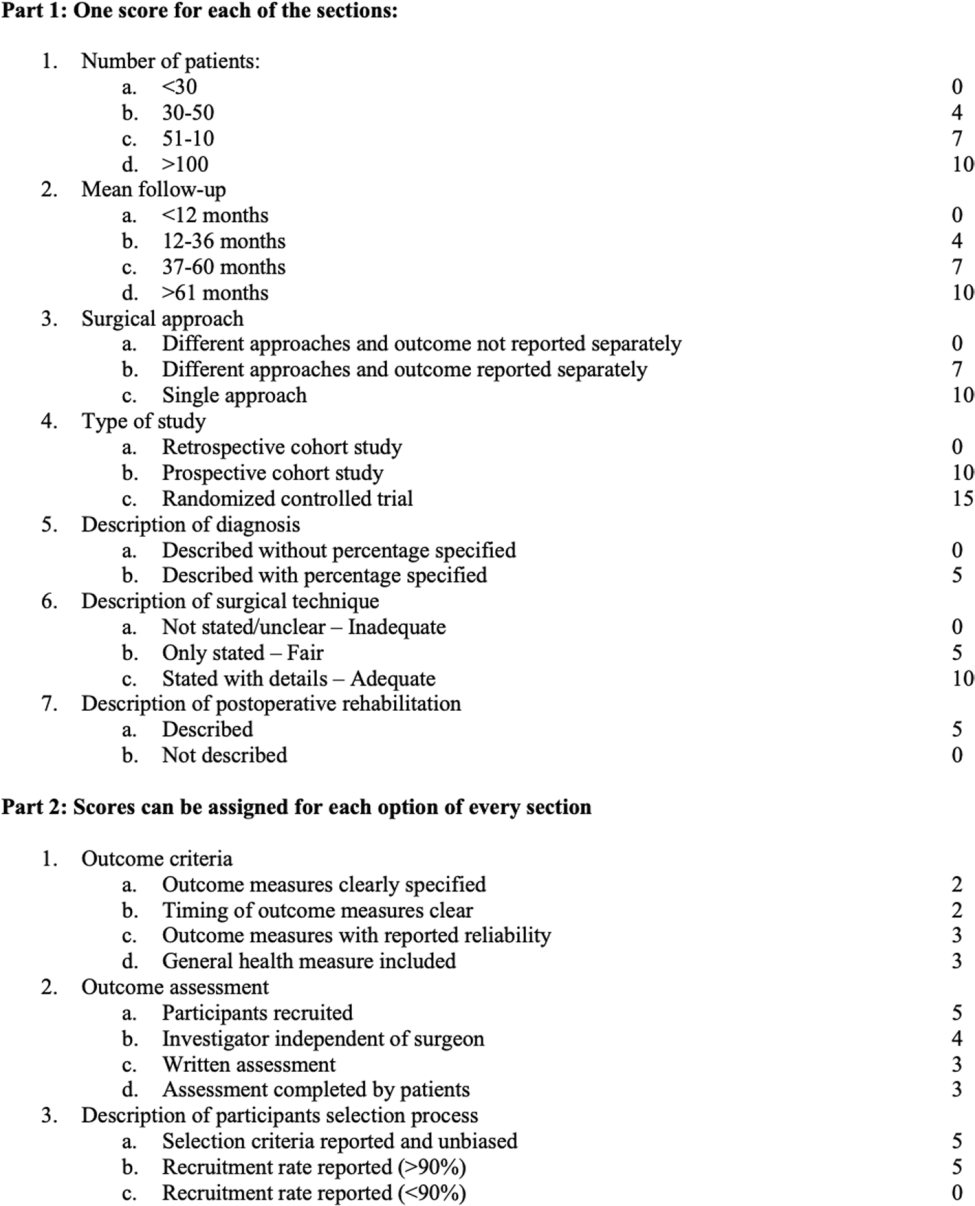
Modified Coleman Methodology Score

Two independent reviewers (F.Ma. and V.D.M.) separately examined all the identified studies and extracted data. During initial review of the data, the following information was collected for each study: title, first author, year of publication, study design, number of patients, patients died and lost at follow-up, age of patients, length of follow-up, indication for index surgery, PFR implant used, complication types, reoperations for any reason, implant loosening, dislocations, deep infections, nerve injuries, and patient-reported outcomes. The level of evidence analysis was determined using the Oxford Centre for Evidence-Based Medicine Levels of Evidence [16]. The methodological quality of each study and the different types of detected bias were assessed independently by each reviewer with the use of Modified Coleman Methodology Score (Fig. 1). The Modified Coleman Methodology score ranges from 0 to 100, with a higher score reflecting higher quality. Final score was categorized as excellent (85-100 points), good (70-84 points), fair (55-69 points), and poor (<55 points). Selective reporting bias was not included in this analysis. Implant failure was defined by need for revision or resection of femoral and/or acetabular component.

Categorical variables were presented as frequency and percentages. Continuous variables were presented as means. A *P*-value <0.05 was considered statistically significant.

## Results

### Study selection

**Fig. 2:**
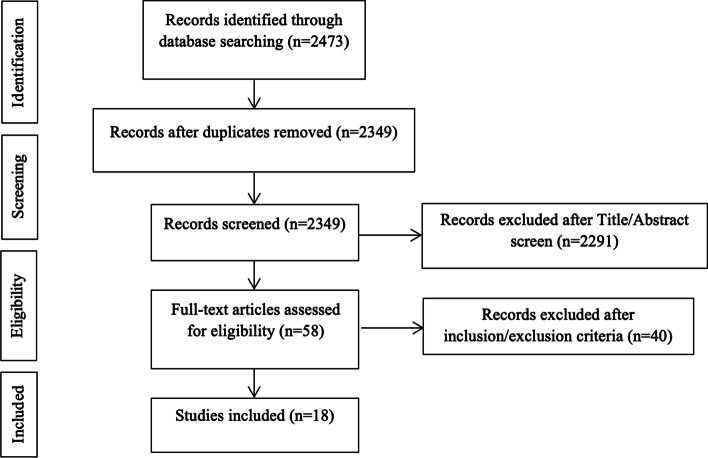
Flow chart of study selection according to PRISMA guidelines for reporting systematic reviews and meta-analyses

The search query resulted in 2473 abstracts that were then examined to determine if they met the inclusion criteria related to the outcome of PFR for primary and revision THA in non-oncologic patients (Fig. 2). Following elimination of duplicate articles, predetermined inclusion and exclusion criteria were applied. In total, 18 articles met the inclusion criteria and were included in the final analysis [3, 4, 17–32] (Table 1). Consensus on which articles would be analyzed in the present study was achieved by discussion between the reviewers based on the predetermined inclusion and exclusion criteria described above.

**Table 1:** Study characteristics and patiens demographics

Author (year)	Study Design, LoE	No. Of Patients	No. Of Hips Initial/Final	Male/Female	Age (range)	Mean Follow-up (years)	MCS
Malkani et al. [17]	Retrospective, IV	49	50/50	18/31	60.6 (27-82)	11.1	42
Haentjens et al. [18]	Retrospective, IV	19	19/19	6/13	78 (63-87)	5	39
Parvizi et al. [3]	Retrospective, IV	48	48/48	16/32	73.8 (42-97)	3	46
Shih et al. [19]	Retrospective, IV	12	13/12	9/3	59 (25-75)	5.7	46
Schoenfeld et al. [20]	Retrospective comparative, III	21	22/19	10/11	76 (62-90)	3.4	33
Bertani et al. [21]	Retrospective, IV	10	10/8	N/A	65 (48-82)	3.7-5.4	41
Gebert et al. [22]	Retrospective, IV	45	45/45	24/21	62 (31-81)	3.2	43
Sewell et al. [23]	Retrospective, IV	15	15/14	8/7	67 (34-85)	5	50
Al Taki et al. [4]	Retrospective comparative, III	63	63/36	25/38	73 (23-94)	3.2	46
McLean et al. [24]	Retrospective, IV	20	20/20	7/13	72 (36-91)	4	41
Dean et al. [25]	Retrospective, IV	8	8/8	4/4	67.5 (50-79)	1.5	39
Colman et al. [26]	Retrospective, IV	21	21/21	N/A	75	1.25	36
Lundh et al. [27]	Retrospective, IV	5	5	4/1	77 (25-91)	4	44
Grammatopoulos et al. [28]	Retrospective, IV	79	80/60	28/52	69 (28-93)	5	39
Viste et al. [29]	Retrospective, IV	44	44/44	13/31	79 (53-97)	6	47
Fenelon et al. [30]	Retrospective, IV	78	79/79	29/49	78.3 (66-90)	2.6	40
De Martino et al. [31]	Retrospective, IV	40	41/41	14/26	64 (29-90)	5	45
Dieckmann et al. [32]	Retrospective, IV	49	49/49	13/36	71 (37-85)	4.3	44
Total	-	626	632/578	228/368	70 (59-79)	4.3 (1.3-11.1)	42 (33-50)

*LoE* Level of Evidence, *N/A* Not Available, *MCS* Modified Coleman Score

### Quality assessment

The quality of the studies was variable, with the average modified Coleman methodology score of the included studies of 42 points (fair, range 33-50 points), showing that the quality of the studies was low. A meta-analysis was not undertaken due to the general poor quality of the studies. (Table 1)

### Demographic data

A total of 626 patients and 632 hips were initially included in this analysis. After excluding 54 hips (8.5%) due to missing data or loss to follow-up, 578 hips with a mean age of 70 years (range, 59-79 years) at the time of surgery were included for the final analysis. The mean follow-up was 4.3 years (range, 1.3-11 years) (Table 1).

### Indication for PFR

All eighteen studies specified the indication, at time of surgery, for use of the PFR, however three cases were not clearly specified (0.5%) [3, 4, 17–32]. Among the studies where details were reported (629 hips), PFRs were most commonly used in revision THA for the treatment of periprosthetic fractures (187 hips, 29.7%), followed by 2-stage revisions with severe bone loss for PJI (184 hips, 29.3%), and aseptic loosening (AL) of previously implanted THA (163 hips, 25.9%). PFRs were used as primary implant in case of comminuted fractures in elderly patients in 10.3% of the cases (65 hips). Other indications including osteosynthesis failure, and non-union were reported in 4.8% of the cases (30 hips). Additional information is further outlined in Table 2.

**Table 2:** Indications for surgery, type of PRF, and method of fixation

Author (year)	Type of implant	Fixation	No. of previous surgery (range)	Primary Fx (rate)	Periprosthetic Fx (range)	AL (rate)	PJI (rate)	Other
Malkani et al. [17]	Monobloc	N/A	N/A	15 (30%)	3 (6%)	26 (52%)	0 (0%)	6 (12%) [5 Girdlestone, 1 arthrodesis]
Haentjens et al. [18]	(Protek A.G., Berne, CH)	Cemented	(1-6)	0 (0%)	0 (0%)	19 (100%)	0 (0%)	0 (0%)
Parvizi et al. [3]	MRS (Stryker Orthopaedics, Mahwah, NJ, US)	Cemented	2.7 (0-8)	1 (2%)	20 (42%)	13 (27%)	13 (27%)	0 (0%)
Shih et al. [19]	Custom-made (United Ustar System, Taipei, Taiwan)	Cemented	6.5 (3-22)	0 (0%)	3 (23%)	3 (23%)	9 (70%)	0 (0%)
Schoenfeld et al.[20]	Modular PFR (Howmedica, Allendale, NJ; Biomet, Warsaw, IN, US)	Cemented	N/A	21 (100%)	0 (0%)	0 (0%)	0 (0%)	0 (0%)
Bertani et al. [21]	JVC-IX (Wright Medical Technology Inc., Arlington, TN, US)	Cemented	N/A	2 (20%)	0 (0%)	8 (80%)	0 (0%)	0 (0%)
Gebert et al. [22]	MUTARS (Implantcast GmbH, Buxtehude, DE)	Cemented (3), Cementless (42)	N/A	0 (0%)	9 (20%)	19 (42%)	16 (36%)	0 (0%)
Sewell et al. [23]	METS (Stanmore Implants worldwide Ltd, Stanmore, UK)	Cemented (2), Cementless (13)	2.8 (1-4)	0 (0%)	2 (13%)	3 (20%)	9 (60%)	1 (7%) [Painful excision arthroplasty]
Al Taki et al. [4]	MRS (Stryker Orthopaedics, Mahwah, NJ, US)	Cemented (33), Cementless (3)	2 (1-5)	0 (0%)	27 (43%)	27 (43%)	7 (13%)	2 (3%) [Instability + bone loss]
McLean et al. [24]	GMRS (Stryker Inc., Mahwah, NJ, US)	Cemented	N/A	4 (20%)	9 (45%)	0 (0%)	7 (35%)	0 (0%)
Dean et al. [25]	METS (Stanmore Implants worldwide Ltd, Stanmore, UK)	N/A	3.1 (1-11)	8 (100%)	0 (0%)	0 (0%)	0 (0%)	0 (0%)
Colman et al. [26]	Modular Endoprosthetic PFR	N/A	N/A	0 (0%)	21 (100%)	0 (0%)	0 (0%)	0 (0%)
Lundh et al. [27]	METS (Stanmore Implants worldwide Ltd, Stanmore, UK)	Cemented (3), Cementless (2)	N/A	0 (0%)	5 (100%)	0 (0%)	0 (0%)	0 (0%)
Grammatopoulos et al. [28]	METS (Stanmore Implants worldwide Ltd, Stanmore, UK)	Cemented	2.4 (0-17)	12 (15%)	16 (20%)	6 (8%)	40 (50%)	4 (5%) [Instability + pseudotumor]
Viste et al. [29]	GMRS (Stryker Inc., Mahwah, NJ, US)	Cemented	2 (1-10)	0 (0%)	15 (34%)	16 (36%)	12 (27%)	1 (2%) [Instability]
Fenelon et al. [30]	GMRS (Stryker Inc., Mahwah, NJ, US); LPS (DePuy Synthes, Warsaw, IN, US)	N/A	1.4 (0-10)	2 (2.5%)	50 (63%)	9 (11%)	5 (6.3%)	13 (16.4%) [2 Instability, 2 Osteoarthritis, 9 Osteosynthesis failure]
De Martino et al. [43]	GMRS (Stryker Inc., Mahwah, NJ, US)	Cemented (37), Cementless (4)	3.6 (1-11)	0 (0%)	7 (17%)	14 (34%)	17 (42%)	3 (7%) [Nonunion]
Dieckmann et al. [32]	MUTARS (Implantcast GmbH, Buxtehude, DE)	Cemented	2.5	0 (0%)	0 (0%)	0 (0%)	49 (100%)	0 (0%)
Total	-	-	3.0 (0-22)	65 (10.3%)	187 (29.7%)	163 (25.9%)	184 (29.3%)	30 (4.8%)

*Fx* Fracture, *N/A* Not Available, *AL* Aseptic Loosening, *PJI* Periprosthetic Joint Infection

Eleven studies (418 of 578 hips, 70.9%) reported the average number of operations before the PFR was performed [3, 4, 18, 19, 23, 25, 28–32], the mean number of procedures was 3.0 (range, 0-22). Type of fixation and implant characteristics are outlined in Table 2.

### Reoperations

The all-cause reoperation-free survivorship after PFR implantation was 76.6% (445 of 578 hips). The overall reoperation rate was 23.4% (135 of 578 hips) at mean follow-up of 4.3 years (range, 1.3-11 years) (Table 3). Dislocation and infection were the most common reasons for reoperation with an incidence of 7.6% (44 of 578 hips) and 6.4% (37 of 578 hips), respectively. The reoperation rate for aseptic loosening of the implant was 5.9% (34 of 579 hips). Reoperation rate due to other complication including periprosthetic fracture and hematoma was 3.1% (19 of 578). Among those, periprosthetic fracture was the most frequent complication requiring surgery in 12 hips (2.1%).

**Table 3:** Complications and reoperations of PFR usage

Author (year)	No. Of Hips	Reoperation (rate)	Revision/Resection (rate)	Dislocation (rate)	Infection (rate)	AL (rate)	Other (rate)
Malkani et al. [17]	50	21 (40%)	16 (32%)	11 (22%) [7 closed reductions, 2 femoral revisions, 2 acetabular revision]	3 (6.3%) [2 DAIR, 1 antibiotic tp]	11 (22.0%) [4 revisions stem, 7 revision cup]	4 (%) [2 hematoma, 1 sciatic nerve palsy, 1 implant fracture - revised]
Haentjens et al. [18]	19	8 (42%)	2 (11%)	7 (36.8%) [5 closed reduction, 2 reoperations]	2 (10.5%) [1 DAIR, 1 revision]	1 (5.26%) [revision cup]	3 (15.8%) [screw loosening]
Parvizi et al. [3]	48	11 (23%)	10 (20%)	8 (16.7%) [6 revisions, 2 closed reductions]	1 (2.1%) [DAIR]	4 (8.3%) [3 revisions cup, 1 resection]	0 (0%)
Shih et al. [19]	12	8 (67%)	7 (58%)	5 (42%) [2 closed reductions, 3 resections]	4 (33%) [1 revision, 3 resections]	1 (8.3%) [revision]	6 (50%) [3 greater trochanter displacement, 1 HO, 2 LLD]
Schoenfeld et a l. [20]	19	3 (16%)	2 (11%)	2 (11%) [closed reductions]	1 (5.2%) [revision]	0 (0%)	5 (26.3%) [2 periprosthetic fractures – 1 osteosynthesis & 1 revision, 1 hardware failure, 2 DVT]
Bertani et al. [21]	8	5 (63%)	4 (50%)	3 (37.5%) [revision]	1 (12.5%) [revision]	0 (0%)	1 (12.5%) [periprosthetic fracture]
Gebert et al. [22]	45	8 (18%)	8 (18%)	1 (2.2%) [revision]	5 (11.1%) [revisions]	2 (4.4%) [revision]	0 (0%)
Sewell et al. [23]	14	3 (21%)	2 (14%)	2 (14.3%) [1 closed reduction, 1 revision]	2 (14.3%) [1 resection, 1 DAIR]	0 (0%)	0 (0%)
Al Taki et al. [4]	36	6 (17%)	5 (14%)	3 (8.3%) [revisions]	1 (2.8%) [DAIR]	2 (2.8%) [1 resection, 1 revision]	0 (0%)
McLean et al. [24]	20	4 (20%)	3 (15%)	3 (15.0%) [1 closed reduction, 2 revisions]	2 (10.0%) [1 DAIR, 1 antibiotic tp]	0 (0%)	1 (5.0%) [periprosthetic fracture – revision]
Dean et al. [25]	8	0 (0%)	0 (0%)	0 (0%)	0 (0%)	0 (0%)	0 (0%)
Colman et al. [26]	21	5 (24%)	5 (24%)	3 (14.3%) [revisions]	2 (19.0%) [revisions]	0 (0%)	N/A
Lundh et al. [27]	5	0 (0%)	0 (0%)	2 (40.0%) [closed reductions]	1 (20.0%) [antibiotic tp]	0 (0%)	0 (0%)
Grammatopoulos et al. [28]	80	17 (21%)	11 (14%)	3 (3.7%) [2 closed reductions, 1 open reduction]	9 (11.2%) [4 DAIR, 4 revisions, 1 antibiotic tp]	3 (3.7%) [1 cup revision, 2 stem revisions]	6 (7.5%) [5 periprosthetic fractures - 2 cup revisions, 2 stem revisions, 1 osteosynthesis, 1 peroneal nerve injury]
Viste et al. [29]	44	9 (20%)	7 (16%)	6 (13.6%) [5 revisions, 1 closed reduction]	2 (5.5%) [1 DAIR, 1 resection]	1 (2.3%) [resection]	1 (2.3%) [wound drainage]
Fenelon et al. [30]	79	4 (5%)	4 (5%)	7 (8.9%) [4 closed reduction, 3 revisions]	3 (3.8%) [antibiotic tp]	1 (1.3%) [revision]	5 (6.3%) [DVT]
De Martino et al. [31]	41	9 (22%)	7 (17%)	2 (4.9%) [revisions]	3 (7.3%) [2 DAIR, 1 revision]	2 (4.9%) [revisions]	2 (4.9%) [periprosthetic fracture - revision]
Dieckmann et al. [32]	49	14 (28.6%)	9 (18.4)	6 (12.2%) [5 open, 1 closed]	2 (4.1%) [revision]	6 (12.2%) [4 cup revision, 2 stem revision]	7 (14.3%) [1 periprosthetic fx – resection, 6 wound complication]
Total		135 (23.4%)	102 (17.6%)	74 (12.8%)	44 (7.6%)	34 (5.9%)	41 (7.1%)

*N/A* Not Available, *DAIR* Debridement Antibiotics Implant Retention, *DVT* Deep Venous Thrombosis, *HO* Heterotopic Ossifications, *LLD* Leg Length Discrepancy, *tp* therapy, *AL* Aseptic Loosening, *PFR* Proximal Femoral Replacement

### Complications

All 18 studies included complications rates [3, 4, 17–32]. The overall complication rate was 27.2% (157 of 578 hips). The most common complication reported was dislocation in 12.8% of the cases (74 of 578 hips), followed by infection in 7.6% (44 of 578 hips), implant aseptic loosening in 5.9% (34 of 578 hips), and periprosthetic fracture in 2.1% (12 of 529 hips). Other complications including nerve injuries, hematoma, wound complications, and deep vein thrombosis (DVT) were reported in 5.0% (29 of 578 hips). Further information is outlined in Table 3.

### Dislocation

Dislocation after PFR insertion was the most frequent post-operative complication observed. The overall prevalence of dislocation was 12.8% (74 of 578 hips) (Table 3). In case of dislocation, conservative treatment with closed reduction was performed in 40.5% (30 of 74 dislocations), and reoperation was required for 59.5%% of all dislocations (44 of 74 dislocations) (Table 3). Among those, revision or resection arthroplasty were performed in 81.8% (36 hips). Open reduction was performed in 6 hips (13.6%) [28], and 2 hips were treated with advancement and reattachment of the greater trochanter and by a firm closure of the fascia lata [18].

### Aseptic loosening, infection, and other complications

The incidence of implant aseptic loosening for either femoral stem or cup was 5.9% (34 of 578 hips). All cases of aseptic loosening reported required further reoperation. Among those, revision of the acetabular component was performed in 47.0% of the cases (16 of 34 loose implants), revision of both components was performed in 20.6% (7 of 34 hips), revision of the femoral component in 23.5% (8 of 34 hips), and resection arthroplasty in 8.8% (3 of 34 hips). The overall reoperation rate due to aseptic loosening of the implant was 5.9% (34 of 578 hips).

The overall incidence of infection was 7.6% (44 of 578 hips), of those, further reoperation was required in 84.0% of the cases (37 hips), whilst 15.9% of the cases (7 hips) were treated conservatively with suppressive antibiotic therapy. Of the infected hips that required surgery, revision or resection arthroplasty was performed in 23 cases (of 37 hips, 62.2%), whilst debridement, antibiotics, and implant retention (DAIR) was performed in 14 cases (of 37 hips, 37.9%) (Table 3). The overall reoperation rate due to infection was 6.4% (37 of 578 hips).

Other complications were reported in 41 cases (of 578, 7.1%). Among those, surgical treatment was required in 46.3% of the cases (19 of 41 complications), and periprosthetic fracture was the most frequent complication that required subsequent surgery (12 hips, 2.1%). In case of periprosthetic fracture, revision of the implant was reported in 75% of the cases (9 out of 12 hips), and osteosynthesis in 25% (3 hips). Complications that did not require surgery were 53.7% (22 hips of 41 complications), and DVT was the most frequent with a reported incidence of 1.2% (7 hips). Further information is outlined in Table 3.

### Clinical scores

Among 18 studies, 10 studies recorded clinical outcomes of PFRs. Six studies reported the preoperative Harris Hip Score (HHS, excellent >90 points, good between 80 to 90 points, fair between 50 to 79 points, and poor <50 points) [3, 17, 19, 22, 23, 29], and 8 studies noted the postoperative HHS [3, 17, 19, 22–24, 29, 32]. The average postoperative HHS was 72.6 (fair; range, 64.9-83 points). In the 6 studies (213 hips) that have both preoperative and postoperative HHS, improvements were seen on the HHS from mean 35.7 points (poor; range, 30-46 points) preoperatively to mean 72.8 points (fair; range, 65.8-78 points) at the latest follow up. Two studies (116 hips) reported a mean postoperative Oxford Hip Score (OHS) of 43.7 points (poor; range, 28-54.9 points) [4, 28]. Two studies [18, 20] reported improvements on the Merle d’Aubigné from mean preoperative 4.4 points (range, 3.8-5.1 points) to mean postoperative 14.5 (range, 12.5-16). Al-Taki et al [4] reported a preoperative Western Ontario and McMaster Universities Arthritis Index (WOMAC) of 49.2 (poor) and a postoperative WOMAC mean of 62.2 (good). Toronto Extremity Salvage Score (TESS) was noted postoperatively in two studies [23, 24]. It is a patient-reported measure of function designed to assess physical disability for patients after limb-salvage surgery for musculoskeletal tumors. Its lower extremity version consists of 30 questions regarding everyday activities such as dressing, working, mobility and leisure and allows a percentage score to be calculated. The mean postoperative score was 69.5% (range 68-71%). Further information on clinical outcomes is outlined in Table 4.

**Table 4:** Clinical outcomes of proximal femoral replacement implants

Author (year)	Preoperative (range)	Postoperative (range)	*P* (value)
Malkani et al. [17]	HHS 46±13 (31-83) Mayo Clinic hip score 30±17 (11-60)	HHS 76±16 (41-94) Mayo Clinic hip score 57±18 (18-75)	<0.01 <0.01
Haentjens et al. [18]	Merle d’Aubigné 5.1	Merle d’Aubigné 14.9	N/A
Parvizi et al. [3]	HHS 37.1 (15-61)	HHS 64.9 (13-91)	<0.05
Shih et al. [19]	HHS 30 (16.-42)	HHS 83 (68-92)	N/A
Schoenfeld et al. [20]	Primary: Merle d’Aubigné N/A Revision: Merle d’Aubigné 3.77	Primary: Merle d’Aubigné 16 Revision: Merle d’Aubigné 12.5	N/A
Bertani et al. [21]	N/A	MSTS 13.8±6.8	N/A
Gebert et al. [22]	HHS 30 (8-63)	HHS 78 (57-95)	N/A
Sewell et al. [23]	HHS 28 (13-49) TESS 26% (14-40)	HHS 69 (39-85) TESS 71% (35-82)	<0.0001 <0.0001
Al Taki et al. [4]	WOMAC 49.2 OHS 34.9 SF-12 physical 30.8 SF-12 mental 38.9 UCLA activity 2.6	WOMAC 62.2 OHS 54.9 SF-12 physiscal 37 SF-12 mental 50.8 UCLA activity 3.9	0.168 0.003 0.220 0.030 0.528
McLean et al. [24]	N/A	SF-36 physical 53 (44-62) SF-36 mental 51 (41-64) TESS 68 (32-98)	N/A
Dean et al. [25]	N/A	HHS 71.4 (64-85)	N/A
Colman et al. [26]	N/A	N/A	N/A
Lundh et al. [27]	N/A	N/A	N/A
Grammatopoulos et al. [28]	N/A	OHS 28 (4-48)	N/A
Viste et al. [29]	HHS 42.8±20 (25.9-82.9)	HHS 65.8±15.6 (21-87.7)	0.0009
Fenelon et al. [30]	N/A	N/A	N/A
De Martino et al. [31]	N/A	N/A	N/A
Dieckmann et al. [32]	N/A	HHS 69 (40-94)	N/A

*MSTS* Musculo-Skeletal Tumor Society score, *TESS* Toronto Extremity Salvage Score, *HHS* Harris Hip Score, *N/A* Not Available, *OHS* Oxford Hip Score, *UCLA* University of California at Los Angeles, *WOMAC* Western Ontario and McMaster Universities, *N/A* Not Available

## Discussion

Our review of the literature suggests that PFR implants are an effective way to manage severe femoral bone loss in non-oncologic primary and revision THA in case of elderly and less active patients with multiple comorbidities where an early mobilization and immediate full weight bearing are crucial for a faster recovery [33]. The all-cause reoperation-free survivorship after PFR implantation was 76.6% (447 of 578 hips) at a mean follow-up of 4.3 years. The overall complication rate was high at 27.2% (157 of 578 hips), with dislocation as the most commonly reported (12.8%), followed by infection (7.6%), and implant AL (5.9%), suggesting that despite acceptable short-to mid-term survivorship given the high complexity of these patients, PFR should be considered as a salvage procedure when other reconstruction options are no longer available. In addition, PFR are currently used in multiple settings, the most common indication was periprosthetic fracture in 29.7% of the cases (187 hips), followed by PJI in 29.3% (184 hips), AL in 25.9% (163 hips), primary comminuted fracture in osteoporotic bone in 10.3% (65 hips), and other indications in 4.8% (30 hips).

Among the current alternative surgical options, allograft prosthesis composites (APC) are usually preferred in case of young patients with primary bone tumors and failed THA where an adequate bone stock is required for potential further revisions. While the megaprostheses are associated with early weight bearing and superior early outcomes, allograft-prosthesis composites have shown improved functional outcome and implant survivorship at long-term follow-up. In addition, they allow the reattachment of the gluteus and iliopsoas tendons supporting hip biomechanics and increasing postoperative hip stability. However, infection, non-union, allograft resorption, periprosthetic fracture and risk of disease transmission continue to be major issues, and the final outcome is strictly related on the etiology, soft tissue damage, type of bone defect, method of reconstruction, and preparation of the allograft [12, 13].

Despite the effectiveness of PFR in restoring function, overall complications rate is high. Hip dislocation was the most frequent complication with an overall prevalence of 12.8 % (74 of 578 hips), higher than the one usually seen after revision THA with conventional implants at short-to-midterm follow-up [34]. Among those, the 59.5% required subsequent reoperation (44 hips), whilst 40.5% were treated nonoperatively (30 closed reductions). In case of reoperation for recurrent dislocation, the majority (81.8%) required a full revision or resection arthroplasty, suggesting the complex management after dislocation in these kinds of patients. However, if excluding the studies that used monobloc and custom-made implants and considering only the ones that implanted modular PFR implants [3, 4, 20–32] the overall dislocation rate was 10.3% (51 of 497 hips), suggesting that modularity enables the surgeon to restore better offset, limb length and soft-tissue tension, providing better postoperative stability compared to older monobloc implants. In addition, our results showed a slightly reduced dislocation rate compared with the rates reported by Vaishya et al [35] that noted a dislocation rate of 14.6% (out of 245 PFRs) at a mean 44 months follow-up in a critical analysis of proximal and distal femoral replacement, and by Korim et al [33] that noted a dislocation rate of 15.7% (out of 356 PFRs) at mean 45 months follow-up in non-oncologic conditions, suggesting that newer implants may provide increased stability. In fact, if we consider only the recent literature (after 2010) [4, 22–32], the dislocation rate results considerably reduced compared to the previous studies probably related not only to the modular systems but also to the increased usage of constrained acetabular liners and dual mobility cups [36–39]. Recurrent dislocation is multifactorial and commonly related to multiple previous procedure and inadequate soft tissue envelop with abductor mechanism deficiency, especially of the posterior vertical fibers of the gluteus medius are considered the main actor in providing dynamic hip stability [40, 41]. In addition, most of the patients included are relatively old with multiple comorbidities that can increase the risk of dislocation [42–44]. Modular implants present a porous-coated proximal surface that promotes osseointegration with the possibility to approximate the retained proximal host bone to the implant enhancing implant’s stability [3]. Moreover, a possible solution to reduce the dislocation rate in case of abductor deficiency was proposed by Du et al. [45]. The authors used a band-shaped artificial ligament wrapped spirally around the proximal site of the total femur prosthesis for periacetabular soft tissue reconstruction in a cohort of 48 PFRs implanted for neoplastic reasons and reported a reduced dislocation rate [46].

Infection was the second most frequent complication reported in 7.6% of the cases (44 of 578 hips), in line with what have been previously reported [32, 34, 47]. Among those, further surgery was required in 84.1% of the cases (37 of 44 infections), while conservative treatment with suppressive antibiotic therapy was reported in 15.9% of the cases (7 of 44 infections). Among the PJIs that underwent subsequent surgery, 14 of them were DAIR (37.8%) and 23 of them were either 2-stage revision to a total femur replacement or resection arthroplasty (62.2%). PJI remains the most challenging complication after PFR because of poor quality soft tissue, poor overall health, and long operative times [24, 48, 49]. Currently, surface coating with iodine and silver [50] have shown a reduced infection rate, improving implant retention and reducing amputations in case of PJI, however, these implants were not clearly used in all the studies included and it was not possible to stratify the infection rate regarding this characteristic [32, 51, 52].

Aseptic loosening of the implant was reported with an incidence of 5.9% (34 of 578 hips), comparing favorably with what has been previously reported [35]. All cases of AL required subsequent reoperation, revision of the cup was performed in 47.1% of the cases (16 hips), followed by revision of the femoral component in 23.4% (8 hips), revision of both components in 20.5% (7 hips), and resection arthroplasty in 8.8% (3 hips).

Despite a relatively high level of complications for current treatment option, patients receiving a PFR showed considerable improvement in a variety of functional scores. Specifically, it was associated with an average of a 42.5 HHS increase between preoperative and postoperative periods. On average, patient improved from “poor” health (mean preoperative HHS of 35.7) to “fair” health (mean postoperative HHS of 72.8) at latest follow-up. These results suggest that PFR remain an efficacious procedure, improving patient functionality and outcomes, especially in complex patients with severe proximal femoral bone loss.

There were a variety of limitations in this study. First, we were limited by the quality of the original studies, the variability in inclusion criteria as well as the methods for reporting the evaluated variables, and number of patients analyzed. Second, our methodology did not allow for identification of unpublished literature on PFR and is limited by potential publication bias. Several different outcome scores were used across the included studies to assess overall hip function. The studies included were heterogeneous, including small sample sizes and different implant used. The studies covered a large time period reflecting the use of variable implant designs from original monobloc to new modular implants. Limited information available on the complications in each cohort did not allow to stratify for indication and provide a better overview on which indication of PFR is associated with better/worse outcomes.

## Conclusion

To date, given the designs available and the current literature, PFR should be considered a valuable option in case of salvage procedure in complex patients affected by severe proximal femoral bone loss when other available reconstruction options cannot be utilized. Newer designs have shown improved stability and clinical outcomes; however, dislocation and infection remain major issues after PFR, and long-term survivorship has not been clearly defined. In conclusion, PFR should be considered as a limb salvage option in carefully selected patients when other options are not feasible.

## Data Availability

All data generated or analyzed during this study are included in this published article. The datasets used and/or analyzed during the current study are available from the corresponding author on reasonable request.

## Abbreviations

PFR: Proximal femoral replacement
THA: Total hip arthroplasty
PJI: Periprosthetic joint infection
AL: Aseptic loosening
HHS: Harris Hip Score
WOMAC: Western Ontario and McMaster Universities
TESS: Toronto Extremity Salvage Score
OHS: Oxford Hip Score
APC: Allograft Prosthesis Composites
DVT: Deep Vein Thrombosis
DAIR: Debridement Antibiotics and Implant Retention
